# Generation of IL-3–Secreting CD4^+^ T Cells by Microbial Challenge at Skin and Mucosal Barriers

**DOI:** 10.4049/immunohorizons.1900028

**Published:** 2019-05-16

**Authors:** Shajo Kunnath-Velayudhan, Michael F. Goldberg, Neeraj K. Saini, Tony W. Ng, Pooja Arora, Christopher T. Johndrow, Noemi Alejandra Saavedra-Avila, Alison J. Johnson, Jiayong Xu, John Kim, Nazanin Khajoueinejad, Christopher D. Petro, Betsy C. Herold, Gregoire Lauvau, John Chan, William R. Jacobs, Steven A. Porcelli

**Affiliations:** * Department of Microbiology and Immunology, Albert Einstein College of Medicine, New York, NY 10461; † Department of Pediatrics, Albert Einstein College of Medicine, New York, NY 10461; ‡ Department of Medicine, Albert Einstein College of Medicine, New York, NY 10461

## Abstract

During Ag priming, naive CD4^+^ T cells differentiate into subsets with distinct patterns of cytokine expression that dictate to a major extent their functional roles in immune responses. We identified a subset of CD4^+^ T cells defined by secretion of IL-3 that was induced by Ag stimulation under conditions different from those associated with previously defined functional subsets. Using mouse models of bacterial and viral infections, we showed that IL-3–secreting CD4^+^ T cells were generated by infection at the skin and mucosa but not by infections introduced directly into the blood. Most IL-3–producing T cells coexpressed GM-CSF and other cytokines that define multifunctionality. Generation of IL-3–secreting T cells in vitro was dependent on IL-1 family cytokines and was inhibited by cytokines that induce canonical Th1 or Th2 cells. Our results identify IL-3–secreting CD4^+^ T cells as a potential functional subset that arises during priming of naive T cells in specific tissue locations.

## INTRODUCTION

Interleukin 3 was first described in 1981 as a lymphokine inducing the expression of 20-α-hydroxysteroid dehydrogenase in cultures of splenic lymphocytes from nude mice (1). Subsequent studies showed that IL-3 is produced predominantly by activated T cells and other immune cells such as mast cells (2) and causes growth and/or proliferation of multiple hematopoietic cells (2). Given its supportive effect on many leukocyte lineages, IL-3 was also known as multi-CSF and was hypothesized to be indispensable for hematopoiesis. However, hematopoiesis was unaffected in mice deficient in IL-3 (3). Instead, these mice were found to have defects in delayed-type hypersensitivity (3) and in immunity to parasites (4). More recent studies have shown that IL-3 has a detrimental role in experimental autoimmune encephalitis and myocarditis (2, 5), lupus nephritis (6), sepsis (7), and blood-stage malaria (8) and a beneficial role in anti-tick immunity (9).

Although CD4^+^ T cells are the predominant source of T cell–derived IL-3, the particular subset or subsets of Th cells that produces IL-3 remains poorly defined (8). A classical study in the field of Th differentiation and specialization by Mosmann et al. (10) reported that both Th1 and Th2 clones expressed IL-3, suggesting that IL-3 is not subset specific. However, given the effect of IL-3 on proliferation of mast cells and basophils, its role in antiparasite immunity, and in potentiation of Th2 immunity, most studies have investigated IL-3 in the context of Th2 immune responses (8, 11). In contrast, we observed that IL-3–producing CD4^+^ T cells were also prominent among CD4^+^ T cells specific to *Mycobacterium bovis* bacillus Calmette-Guerin (BCG), which has generally been associated with priming of strong Th1 responses (12). This finding was surprising because IL-3 is seldom studied in the context of mycobacterial immunity and motivated us to further explore this finding. In addition, because most previous work on IL-3–producing CD4^+^ T cells has been performed with in vitro–derived T cell clones, we were motivated to characterize IL-3–secreting CD4^+^ T cells generated under more physiologic conditions.

In this study, we present results suggesting that IL-3–secreting CD4^+^ T cells represent a discrete subset of Th cells arising under particular conditions of T cell priming. Mouse infection models using BCG or HSV-2 showed that cutaneous infection with these microbes led to the generation of IL-3–producing CD4^+^ T cells, whereas i.v. infections did not. In addition, IL-3–producing CD4^+^ T cells were induced by oral infection with *Listeria monocytogenes* or vaginal infection with HSV-2, suggesting that they also arise from introduction of Ags at the mucosal barriers. The IL-3–producing CD4^+^ T cells typically coexpressed GM-CSF and other cytokines that define multifunctionality, and in vitro studies demonstrated that they were generated in the presence of IL-1 family cytokines combined with blockade of cytokines that drive Th1 and Th2 differentiation. The characteristic cytokine expression pattern of these cells, their dependence on initial stimulation by Ags introduced at cutaneous or mucosal barriers, and the unique cytokine milieu driving their generation suggest that IL-3–secreting CD4^+^ T cells are a distinct functionally specialized subset of Th cells.

## MATERIALS AND METHODS

### Mice

Six- to eight-week-old female wild-type (WT) C57BL/6 mice were obtained from The Jackson Laboratory. C57BL/6-P25 TCR– transgenic (Tg) and GFP-expressing C57BL/6–OT-II TCR-Tg mice were bred in our facility from founders obtained from The Jackson Laboratory and G. Lauvau (Albert Einstein College of Medicine, Bronx, NY), respectively. All mice were maintained in specific pathogen-free conditions. All procedures involving the use of animals were in compliance with protocols approved by the Einstein Institutional Animal Use and Biosafety Committees.

### Infection with M. bovis BCG

*M. bovis* BCG-Danish was obtained from Statens Serum Institute (Copenhagen, Denmark) and was grown in Middlebrook 7H9 medium (Difco Laboratories, BD Diagnostic Systems, Sparks, MD) with oleic acid–albumin–dextrose–catalase (OADC Enrichment; Difco Laboratories, BD Diagnostic Systems) and 0.05% tyloxapol (Sigma-Aldrich, St. Louis, MO) (13). Bacteria were grown from low passage number frozen stocks, cultured to midlog phase, and then frozen in medium with 5% glycerol at −80°C. Bacteria were thawed, washed, resuspended in PBS containing 0.05% Tween-80, and sonicated to obtain a single-cell suspension prior to infection. Mice were vaccinated with 2 × 10^6^ CFU of bacteria s.c. at the base of the tail or i.v. in the tail vein. Mice were euthanized 4 wk after vaccination to isolate spleen and draining lymph nodes. Splenocyte and lymph node single-cell suspensions were prepared by gently forcing spleen through a 70-μm cell strainer. RBC lysis step was performed with splenocyte suspension using RBC lysing buffer (Hybri-Max; Sigma-Aldrich).

### Infection with M. tuberculosis

All experiments involving *M. tuberculosis* were conducted in biosafety level 3 conditions. *M. tuberculosis* (strain H37Rv) were cultured at 37°C in Middlebrook 7H9 medium containing 10% (v/v) oleic acid–albumin–dextrose–catalase, 0.5% (v/v) glycerol, and 0.05% (v/v) tyloxapol. Bacteria were grown from low passage number frozen stocks, cultured to midlog phase, and then frozen in medium with 5% glycerol at −80°C. Bacteria were thawed, washed, resuspended in PBS containing 0.05% Tween-80, and sonicated to obtain a single-cell suspension for OD measurements. The final bacterial suspension, which contained 2 × 10^6^ CFU/ml of bacteria in PBS containing 0.05% (v/v) Tween-80 and 0.05% (v/v) antifoam Y-30 (Sigma-Aldrich), was loaded into a nebulizer attached to an airborne infection system called Einstein Contained Aerosol Pulmonizer (14). Mice were exposed to aerosolized H37Rv for 20 min, and ⁓100 H37Rv bacteria were deposited into the lungs of each animal. The inoculum dose was confirmed by plating of whole-lung homogenates at 24 h postaerosol exposure. Four weeks post-infection, mice were euthanized, and mediastinal lymph nodes were harvested for further processing.

### Infection with HSV 2

Both vaginal and skin infections were performed with a clinical isolate, HSV-2 (4674) (15), as described previously (16). Briefly, the virus was propagated on Vero cells (green monkey kidney cell line, CCL-81; American Type Culture Collection), which were passaged in DMEM supplemented with 10% FBS (Atlanta Biologicals). To make frozen stocks, cells were pelleted and lysed by three cycles of freeze thawing. Resulting viral stocks were stored at −80°C and diluted in PBS on the day of infection. For vaginal infections, each mouse was infected with 5 × 10^4^ PFU, which corresponds to a lethal dose in 90% of infected mice (LD90), and for skin infections, each mouse was infected with five times the LD90. For the skin scarification model, mice were depilated on the right flank with a commercial hair removal cream (Nair, Church and Dwight) and allowed to rest for 24 h. Depilated mice were anesthetized with isoflurane (Isothesia, Henry-Schein), abraded on the exposed skin with a disposable emery board for 20–25 strokes, and subsequently challenged with 5 μl of viral suspension deposited on the abraded skin. Mice were then anesthetized for an additional 5 min to allow the virus inoculum to dry. For intravaginal infection, mice were injected with 2.5 mg of medroxyprogesterone acetate (Sicor Pharmaceuticals) s.c. at the scruff of the neck. Five days later, they were anesthetized with isoflurane, and 20 μl of viral suspension was deposited in the vagina using a blunt pipette tip. After infections, the mice were monitored for 7 d for signs of disease. If the mice developed hind-limb paralysis or became morbid, mice were euthanized and organs were not procured (16). Surviving mice were euthanized on day 7 to isolate spleen and draining lymph nodes.

### Infection with L. monocytogenes

*L. monocytogenes* strain 10403s expressing the gene encoding the OVA model Ag was obtained from G. Lauvau (Albert Einstein College of Medicine, Bronx, NY). The bacterial stocks were prepared from clones grown from organs of infected mice and kept at −80°C. High-dose gastric infection was performed as previously described with some modifications (17). Briefly, the cultures were grown overnight in broth heart infusion medium (Sigma-Aldrich), and the next day, fresh cultures were grown until reaching an OD_600_ of 0.8 U (OD_600_ of 0.1 corresponds to 2 × 10^8^ *Listeria*/ml). The cells were then pelleted and diluted in PBS appropriately. The mice were infected by gastric intubation with 200 μl of bacterial suspension, which corresponds to 5 × 10^9^ *Listeria* bacilli. The infecting dose was confirmed by plating dilutions of the inoculum on broth heart infusion agar plates. The mice were monitored for disease and euthanized on day 9 when spleens were isolated.

### Differentiation of CD4^+^ T cells in vitro

For in vitro differentiation of WT CD4^+^ T cells, 25,000 CD4^+^ T cells enriched by negative selection (Stem Cell Technologies) were cultured in a 96-well plate coated with anti-CD3 (clone 145–2C11) and anti-CD28 (clone 37.51) Ab. IMDM medium (Life Technologies) supplemented with penicillin-streptomycin (Life Technologies), FBS (10%; Atlanta Biologicals), β-mercaptoethanol (Life Technologies), and essential and nonessential amino acids (Life Technologies) was used for all in vitro experiments. The medium used for Th1 differentiation contained IL-12 (10 ng/ml) and anti–IL-4 (clone 11B11, 10 μg/ml). The medium used for Th2 differentiation contained IL-4 (10 ng/ml), anti–IFN-γ (IFN-γ, clone XMG1.2, 10 μg/ml), and anti–IL-12 (clone C17.8, 10 μg/ml). The medium used for Th17 differentiation contained anti–IFN-γ (10 μg/ml), anti–IL-4 (10 mg/ml), TGF-β (5 ng/ml), IL-6 (100 ng/ml), and IL-23 (20 ng/ml). The medium used for induced regulatory T (iTreg) differentiation contained IL-2 (5 ng/ml) and TGF-β (5 ng/ml). For Th1 and Th2 differentiation, replenishing media used on day 3 and day 4 contained IL-2 (10 ng/ml). All cytokines were obtained from Peprotech, and Abs were obtained from Bio X Cell. On day 5, cells were harvested for restimulation and subsequent staining. For differentiation of Ag-specific CD4^+^ T cells from TCR-Tg mice, 25,000 CD4^+^ T cells purified by negative selection were cultured with 75,000 irradiated, T cell– depleted WT splenocytes in the presence of cognate peptide (for P25 TCR-Tg cells, FQDAYNAAGGHNAVF; for OT-II TCR-Tg cells, ISQAVHAAHAEINEAGR, both from Mimotopes and used at 5 μg/ml). All cytokines used in the screen to enrich IL-3–secreting cells were used at a concentration of 10 ng/ml except IL-33, which was used at a concentration of 100 ng/ml.

### T cell stimulation, intracellular cytokine staining, and flow cytometry

The splenocytes or lymph node suspensions from vaccinated or infected mice and CD4^+^ T cells differentiated in vitro were restimulated with appropriate Ags for 6 h, with the last 4 h in the presence of monensin (5 μM; Sigma-Aldrich) and brefeldin A (5 μg/ml; Sigma-Aldrich). Restimulation was performed in RPMI medium (Life Technologies) supplemented with HEPES (Life Technologies), penicillin-streptomycin (Life Technologies), FBS (10%; Atlanta Biologicals), β-mercaptoethanol (Life Technolo-gies), essential amino acids (Life Technologies), and nonessential amino acids (Life Technologies). To restimulate single-cell suspensions from mice vaccinated with BCG, mycobacterial lysate prepared as previously described (18) was used at a concentration of 50 μg/ml protein. To restimulate single-cell suspensions from mice infected with *M. tuberculosis*, ESAT-6 peptide (MTEQQWN FAGIEAAASAIQG, Mimotopes) was used at 5 μg/ml. To restimulate single-cell suspensions from mice infected with HSV-2, UV-inactivated HSV-2 was used in a ratio of 5 UV-HSV for 1 splenocyte (19). To restimulate single-cell suspensions from mice infected with *Listeria*, a mixture of OVA and listeriolysin O (LLO) peptides (OT-II peptide, FQDAYNAAGGHNAVF; LLO peptide, NEKYAQAYPNVS; both from Mimotopes and used at 5 μg/ml) were used. Restimulation of in vitro–differentiated T cells was performed with PMA (1 μg/ml; Sigma-Aldrich) and ionomycin (1 μg/ml; Sigma-Aldrich). After re-stimulation, cells were suspended in PBS and stained with viability dye (LIVE/DEAD Fixable Blue Dead Cell Stain; Molecular Probes) and subsequently with fluorochrome-conjugated Abs against surface markers in PBS containing FBS (2%) and sodium azide (0.05%; Sigma-Aldrich). Cells were fixed using paraformaldehyde (2% in PBS; Electron Microscopy Sciences), permeabilized (Fixation/ Permeabilization buffer; eBioscience), and stained for intracellular cytokines. Intracellular cytokine staining buffer used was Dulbecco PBS with calcium and magnesium (Life Technologies) containing FBS (2%), sodium azide (0.05%), HEPES (1%), and saponin (0.1%; Sigma-Aldrich). The cells were then washed with intracellular cytokine staining buffer and PBS before collecting data using a BD LSR-II flow cytometer (BD Biosciences). The following Abs were used for staining: CD4-allophycocyanin-Cy7 (clone RM4–5; Tonbo Biosciences), CD44-eFluor 450 (clone IM7; eBioscience), CD8α-PE-Cy5 (clone 53–6.7; Tonbo Biosciences), B220-PE-Cy5 (clone RA3–6B2; BD Biosciences), MHC class II (MHC II)–PE-Cy5 (clone M5/ 114.15.2; eBioscience), IFN-γ–Alexa Fluor 700 (clone XMG1.2; BD Biosciences), TNF–Alexa Fluor 488 (clone MP6-XT22; BD Biosciences), IL-2–PE-Cy7 (clone JES6–5H4; eBioscience), GM-CSF (clone MP1–22E9; eBioscience), IL-4 (clone 11B11; BD Biosciences), IL-13 (clone eBio13A; eBioscience), IL-17 (clone eBio17B7; eBioscience), IL-10 (clone JES5–16E3; BioLegend), and IL-3–PE (clone MP2–8F8; BD Biosciences).

### Statistical analysis

For individual pairwise comparisons, Student *t* tests were performed to assess the level of statistical significance and were reported as significant for *p* values <0.05. For multiple comparisons, a one-way ANOVA was performed initially to test for significance of overall difference among group means. For *p* values <0.05, post hoc comparisons were made between two groups as indicated in the figure legends. The *p* values of post hoc comparisons were corrected for multiple testing using Dunnett method and were reported as significant for *p* values <0.05. All statistical analyses were performed using GraphPad Prism software, version 7.

## RESULTS

### Induction of IL-3–producing CD4^+^ T cells in vivo

**FIGURE 1:**
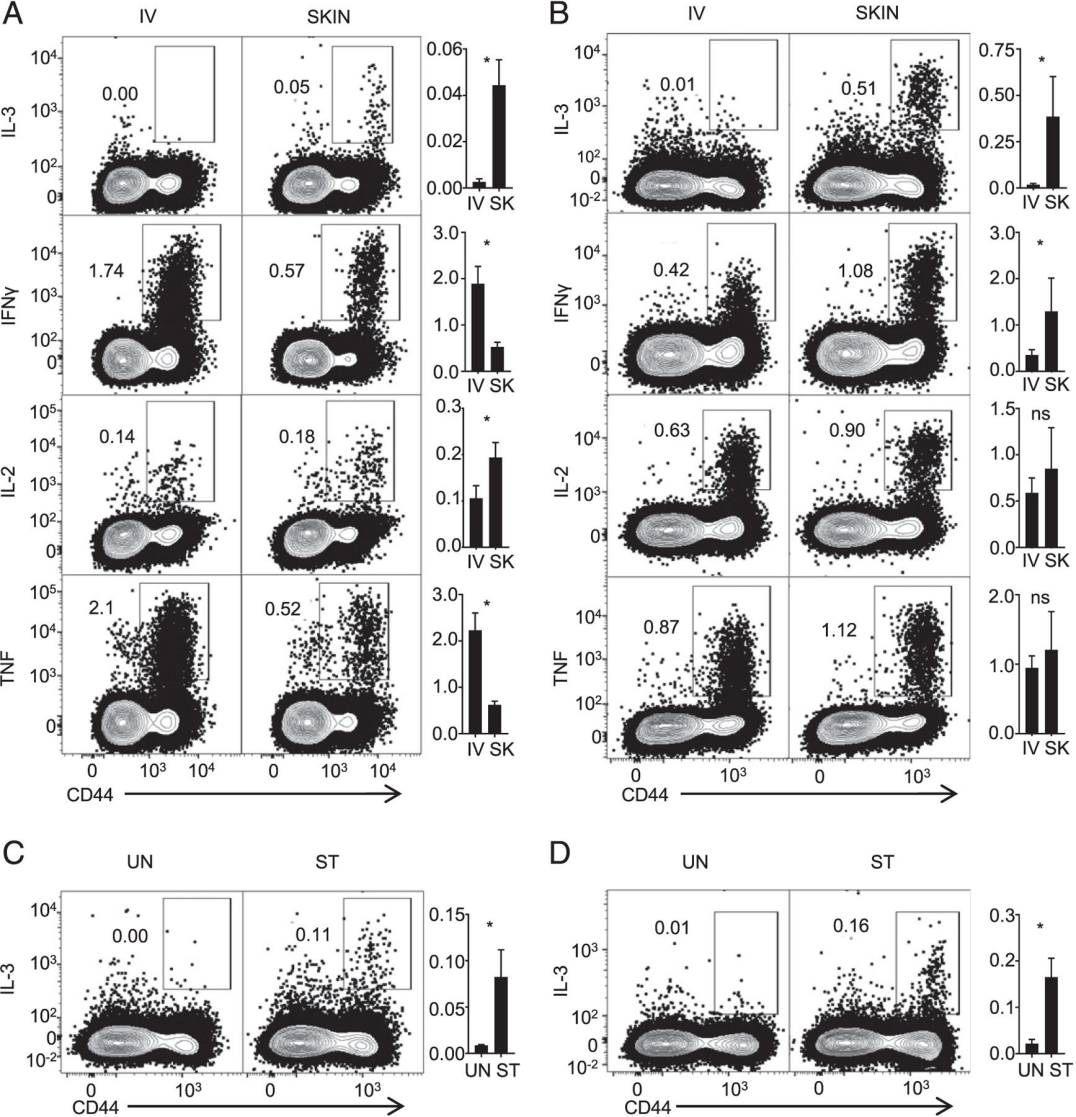
Induction of IL-3–secreting CD4^+^ T cells. **(A)** Mice were infected with *M. bovis* BCG by cutaneous or i.v. injections. Four weeks later, isolated splenocytes underwent stimulation with *M. tuberculosis* lysate and intracellular cytokine staining. Representative contour plots show singlet events from the lymphocyte gate (forward scatter [FSC]/ side scatter [SSC]) that are positive for CD4 and negative for LIVE/DEAD stain, CD8α, B220, and MHC II. Numbers in the contour plots are the percentage of events within the rectangular gates. Bar graphs show the percentage of CD4^+^ T cells that secrete the cytokine shown on the left (skin [SK]). The figure represents results of two independent experiments. **(B)** Mice were infected with HSV-2 cutaneously after skin scarification or by i.v. injection. Seven days later, isolated splenocytes underwent intracellular cytokine staining after restimulation with UV-inactivated HSV-2. The cells were then processed, and the results are displayed as in (A). The figure represents results of two independent experiments. **(C)** Mice were infected with HSV-2 by intravaginal route. Seven days later, splenocytes were isolated and cultured either with (stimulated [ST]) or without (unstimulated [UN]) UV-inactivated HSV-2. The cells were then processed, and the results are displayed as in (A). The figure represents results of three independent experiments. **(D)** Mice were infected with *L. monocytogenes* expressing OVA by oral route. Nine days later, splenocytes were isolated and cultured either with (ST) or without (UN) a mixture of LLO and OVA peptides. The cells were then processed, and the results are displayed as in (A). This experiment was done once. *p < 0.05 for comparisons between i.v. and SK or UN and ST (Student *t* test). ns, not significant.

In our previous work, we have shown that IL-3–producing CD4^+^ T cells develop following vaccination with BCG by s.c. route or infection with *M. tuberculosis* by aerosol (12). We tested the possibility that i.v. BCG infection might induce a higher number of IL-3–secreting CD4^+^ T cells, potentially contributing to the greater protective effects attributed to BCG vaccination by this route (20). Splenocytes from C57BL/6 mice that were vaccinated previously with BCG s.c. or i.v. were stimulated ex vivo with *M. tuberculosis* lysate and stained for intracellular IL-3. Consistent with previous findings, a small population of CD4^+^ T cells from s.c. vaccinated mice produced IL-3 (Fig. 1A, Supplemental Fig. 1A). However, we did not detect IL-3 production from CD4^+^ T cells from mice vaccinated by the i.v. route. The lack of IL-3 production was not due to a generalized reduction in cytokine production because distinct populations of CD4^+^ T cells producing IFN-γ, IL-2, and TNF were visualized after i.v. vaccination (Fig. 1A).

To test whether the effect of route of infection observed above was also seen in a virus infection model, we examined generation of IL-3–producing CD4^+^ T cells following skin infection with HSV-2 (16) compared with i.v. infection with this virus. One week postinfection, splenocytes from infected mice were restimulated with UV-inactivated HSV-2 and then analyzed for intracellular IL-3 by flow cytometry. Similar to the results with BCG infection, we observed a population of IL-3–secreting CD4^+^ T cells in mice infected cutaneously but not in mice infected by the i.v. route (Fig. 1B). In contrast, both routes of infection induced populations of CD4^+^ T cells producing IFN-γ, IL-2, and TNF (Fig. 1B).

Given that mucosal surfaces share with skin the function of acting as barriers to microbes and may provide similar microen-vironments for influencing T cell differentiation, we next tested whether IL-3–producing CD4^+^ T cells were induced in models of mucosal infections. We infected mice with HSV-2 intravaginally (16) and harvested splenocytes 7 d postinfection for restimulation ex vivo with UV-inactivated HSV-2 followed by intracellular IL-3 staining. In another model of mucosal infection, we used intragastric infection with *L. monocytogenes* and 9 d following infection examined splenic T cells for Ag-specific production of IL-3 by intracellular cytokine staining (21). In both mucosal infection models, we detected a distinct population of IL-3–producing CD4^+^ T cells (Fig. 1C, 1D). Collectively, these results suggest that generation of IL-3–secreting CD4^+^ T cells in vivo is restricted to infection or vaccination through barrier surfaces, such as skin and mucosa. Based on our findings, and consistent with previously published results (22), this route dependence for inducing IL-3–secreting CD4^+^ T cells appears to be preserved across a range of different pathogen challenges, including bacteria, viruses, and parasites.

### Coexpression of multiple cytokines by IL-3–producing CD4^+^ T cells

**FIGURE 2:**
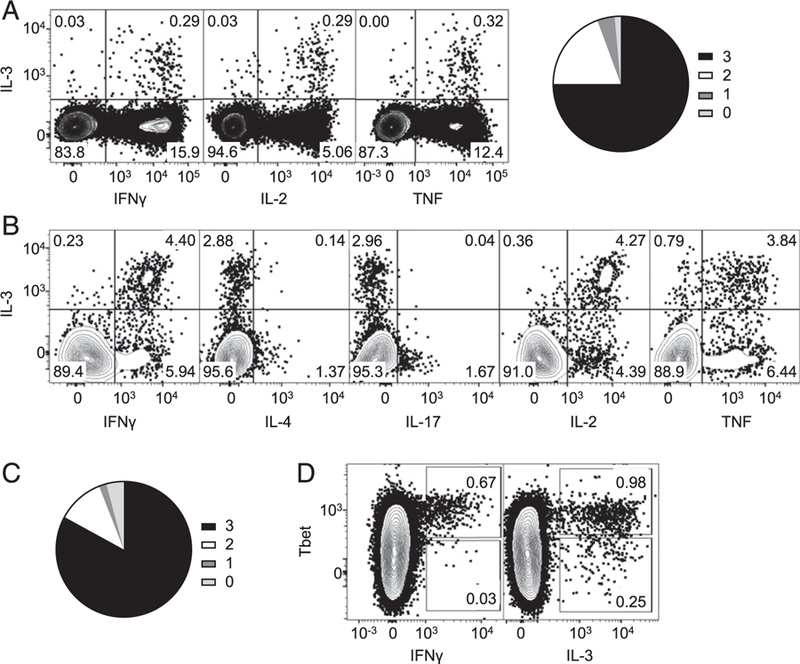
IL-3–secreting CD4^+^ T cells are multifunctional. **(A)** Splenocytes from mice injected with *M. bovis* BCG cutaneously underwent stimulation with *M. tuberculosis* lysate and intracellular cytokine staining. The contour plots represent Ag-experienced CD4^+^ T cells expressing IL-3 and indicated cytokines. Cells shown are singlet events from the lymphocyte (FSC/SSC) gate that are positive for CD4 and CD44 and negative for LIVE/DEAD stain, CD8α, B220, and MHC II. Pie chart depicts the fraction of IL-3–secreting Ag-experienced CD4^+^ T cells that also secrete cytokines that define multifunctionality. Each shade represents number(s) of additional cytokine(s) secreted. The figure represents results of three independent experiments. **(B)** Splenocytes from mice infected with HSV-2 by cutaneous route underwent stimulation with UV-inactivated HSV-2 and intracellular cytokine staining. The contour plots represent Ag-experienced CD4^+^ T cells that are CD44^+^ and expressing IL-3 and indicated cytokines. The figure represents results of two independent experiments. **(C)** Further analyses of the data represented in (B). Pie chart depicts the fraction of IL-3–secreting Ag-experienced CD4^+^ T cells that are CD44^+^ and also secrete cytokines that define multifunctionality. Each shade represents number of additional cytokine(s) secreted. **(D)** Experiments were performed as in (B). Splenocytes were stained for Tbet in addition to intracellular cytokines. Contour plots show single events from the lymphocyte gate that are positive for CD4 and negative for LIVE/DEAD stain, CD8α, B220, and MHC II. Numbers in the rectangular gates are the percentage of events within the corresponding gates. The figure represents results of two independent experiments.

Our previous experiments with BCG-vaccinated mice have shown that IL-3–producing CD4^+^ T cells coexpress IFN-γ, the signature cytokine of Th1 cells, but segregate from cells that produce the canonical Th2 cytokine IL-4 or Th17 cytokine IL-17A (12). Coexpression of IL-3 with IFN-γ was also noted for CD4^+^ T cells from mice infected with virulent *M. tuberculosis* by aerosols (Supplemental Fig. 2A). We next tested in the BCG model whether IL-3 is coexpressed with IL-2 or TNF, as multifunctional Th1 cells express these two cytokines in addition to IFN-γ (23). The results showed that most IL-3–secreting T cells coexpressed either IL-2 or TNF (Fig. 2A). Quantification of coexpression patterns showed that ⁓75% of the IL-3–secreting cells expressed all three cytokines that define multifunctionality (Fig. 2A). In addition, consistent with previous observations (23), IL-3^+^ T cells had higher levels of individual cytokines compared with IL-3–negative T cells (Supplemental Fig. 2B). We also noted that IL-3 secretion did not generally define multifunctional T cells because only a small fraction of multifunctional T cells (⁓5%) coexpressed IL-3. Similar coexpression profiles were also seen with IL-3–secreting CD4^+^ T cells in the HSV-2 skin infection model (Fig. 2B, 2C, Supplemental Fig. 2C), including positivity for Tbet (Fig. 2D), as previously reported with *M. bovis* BCG model (12). Collectively, these results suggest that in these models, IL-3–secreting T cells are closely related to conventionally defined multifunctional Th1 cells and may define a specific subset or activation state of these.

### IL-3 production by canonical T helper populations generated in vitro

**FIGURE 3:**
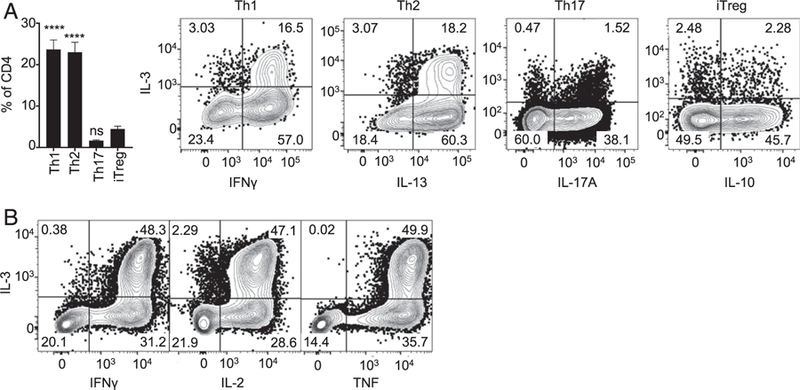
IL-3 production by canonical T helper populations generated in vitro. **(A)** CD4^+^ T cells from naive C57BL/6 mice were purified and cultured on plates coated with anti-CD3 and CD28 Abs in the presence of cytokines and blocking Abs to obtain various T helper populations. On day 5 of culture, cells were stimulated with PMA and ionomycin and subsequently underwent intracellular cytokine staining. During analysis of the flow cytometry data, CD4^+^ T cells were identified as single cells from the lymphocyte gate (FSC/SSC) that are positive for CD4 and negative for LIVE/DEAD stain, CD8α, B220, and MHC II. The bar plot depicts the percentages of IL-3–secreting CD4^+^ T cells in each polarization condition. The contour plots depict coexpression of IL-3 with the signature cytokines for populations differentiated under conditions that promote Th1, Th2, Th17, or iTreg subsets. The *p* value for one-way ANOVA was <0.0001. The *p* values of post hoc tests that compare other groups to iTreg group are provided above each bar after correcting for multiple testing; ****p = 0.0001; ns, not significant. The figure represents results of two independent experiments. **(B)** Splenocytes from OT-II TCR-Tg mice were cultured in the presence of OVA peptide (OVA_323–339_), IL-12, and Ab to IL-4 to generate Th1 cells. Gating strategy as in (A). Contour plots representing coexpression of IL-3 with cytokines that define multifunctionality are shown. The figure represents results of three independent experiments.

Although studies have examined IL-3 expression by T cell clones, no studies, to our knowledge, have examined IL-3 production following activation and differentiation of primary naive CD4^+^ T cells in vitro under defined culture conditions. Such studies are useful for defining the environmental cues and signals that guide differentiation of naive T cells into specific functional subsets (24, 25). Using established in vitro T cell differentiation protocols (26–28), we first tested which of the currently well-defined functional T cell subsets express IL-3. We isolated CD4^+^ T cells from splenocytes collected from naive C57BL/6 mice and stimulated them in vitro with plate-bound CD3 and CD28 Abs under culture conditions known to induce Th1, Th2, Th17, and iTreg cells (26–28). After 5 d of culture, the cells were restimulated with PMA and ionomycin and then underwent intracellular cytokine staining. For each of the culture conditions, we observed robust expression of corresponding signature cytokines (Supplemental Fig. 3A). Of note, we pursued IL-13 as a marker of Th2 polarization because this cytokine was more strongly expressed than IL-4 in our experimental system (Supplemental Fig. 3A). When examined for IL-3 expression, we found that approximately a quarter of cells cultured under Th1 (⁓24%) and Th2 (⁓23%) conditions expressed IL-3, whereas this fraction was small for Th17 (2%) and iTreg (4%) conditions (Fig. 3A). All IL-3–secreting cells derived under the Th1 conditions coexpressed IFN-γ, and those from Th2 conditions coexpressed IL-13 (Fig. 3A). Half of IL-3–producing cells coexpressed IL-17 under Th17-priming condition, whereas no definite coexpression was seen between IL-3 and IL-10 in iTreg-promoting conditions (Fig. 3A). These results were compatible with IL-3 being expressed by a substantial fraction of both Th1 and Th2 cells in vitro (10).

We also generated Th1 cells in vitro from TCR-Tg mice with specificity toward OVA or Ag 85B of *M. tuberculosis*. In these experiments, purified CD4^+^ T cells from TCR-Tg mice were stimulated with cognate peptides using irradiated T cell–depleted splenocytes as Ag-presenting cells. After 5 d of culture under Th1 conditions, the cells underwent stimulation with PMA and ionomycin and intracellular cytokine staining. CD4^+^ T cells from both Tg mice produced IFN-γ as expected, and a portion of them expressed IL-3 (Fig. 3B, Supplemental Fig. 3B). Similar to Th1 cells generated from WT mice, IL-3–secreting cells coexpressed IFN-γ, IL-2, and TNF (Fig. 3B, Supplemental Fig. 3B). These results further supported the idea that IL-3–secreting cells were closely related to multifunctional Th1 cells.

### Cytokines of the IL-1 family favor generation of IL-3^+^ CD4^+^ T cells

Although IL-3 was coexpressed with signature cytokines of Th1, Th2, and to a certain extent Th17 cells, the dependence of IL-3–producing T cells on the route of Ag exposure in vivo indicated unique conditions for their induction and suggested that it might be possible to generate relatively pure IL-3–producing cells by mimicking these conditions in vitro. Based on this assumption, we devised an approach involving the culturing of naive CD4^+^ T cells in the presence of Abs that neutralize Th1 and Th2 cytokines with addition of cytokines that are known to be strongly associated with skin and mucosal barriers, including IL-1α, IL-1β, IL-33 and thymic stromal lymphopoietin (29–32). We also included IL-18, which has been shown to induce cells that secrete IFN-γ and IL-3 (33), and IL-7, which has been shown to enhance in vitro generation of GM-CSF–producing cells that coexpress IL-3 (34). In addition, we included IL-3 and GM-CSF to test if these related cytokines have an autocrine effect, as in the case of IL-4 for Th2 differentiation (25).

**FIGURE 4:**
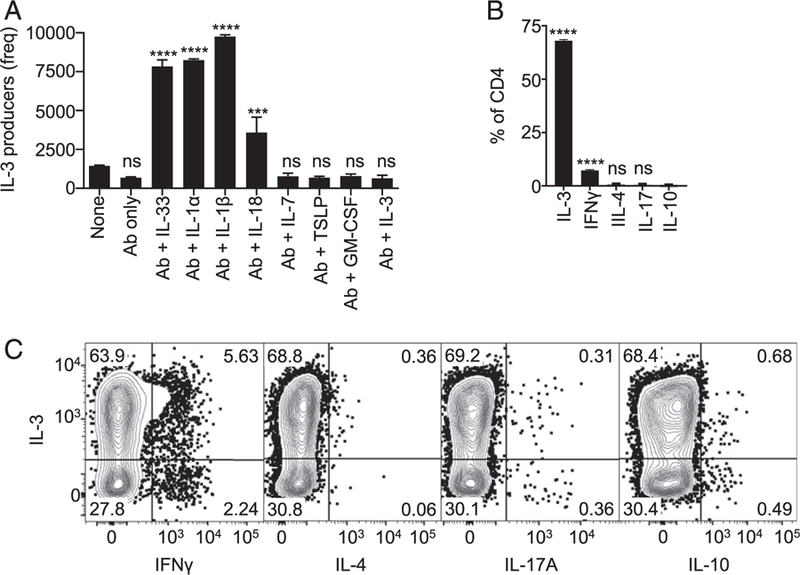
Members of IL-1 family of cytokines favor the generation of IL-3–secreting CD4^+^ T cells in vitro. **(A)** CD4^+^ T cells from naive C57BL/6 mice were purified and cultured on plates coated with anti-CD3 and CD28 Abs in the presence of indicated cytokines and blocking Abs to IFN-γ, IL-12, and IL-4. On day 5 of culture, cells were stimulated with PMA and ionomycin followed by intracellular cytokine staining. During analysis of the flow cytometry data, CD4^+^ T cells were identified as single cells from the lymphocyte gate (FSC/SSC) that are positive for CD4 and negative for LIVE/DEAD stain, CD8α, B220, and MHC II. Absolute numbers of IL-3–secreting CD4^+^ T cells in each condition are shown. Ab indicates mixture of Abs to IFN-γ, IL-12, and IL-4. The *p* value for one-way ANOVA is <0.0001. The *p* values of post hoc tests that compare other groups to control group with no Ab or cytokines added (none) are provided above each bar after correcting for multiple testing. ****p = 0.0001, ***p = 0.0008. ns, not significant. The figure represents results of two independent experiments. **(B)** Splenocytes from OT-II TCR-Tg mice were cultured in the presence of OVA_323–339_ peptide, IL-1α, and blocking Abs to IFN-γ, IL-12, and IL-4. On day 5 of culture, cells were stimulated with PMA and ionomycin followed by intracellular cytokine staining. Gating strategy as in (A). The bar graph depicts the percentage of CD4^+^ T cells secreting each cytokine. The *p* value for one-way ANOVA is <0.0001. The *p* values of post hoc tests that compare other groups to IL-10 group are provided above each bar after correcting for multiple testing. ****p = 0.0001. ns, not significant. The figure represents results of two independent experiments. **(C)** Further analyses of the data represented in (B). Contour plots of CD4^+^ T cells representing coexpression of IL-3 with cytokines characteristic of known T helper subsets are shown.

For the initial experiments, WT splenocytes were stimulated with plate-bound CD3 and CD28 Abs and cultured for 5 d in the presence of blocking Abs (a mixture of neutralizing Abs to IL-4, IL-12, and IFN-γ) and one of the selected cytokines. The cells then underwent restimulation with PMA and ionomycin and sub-sequently intracellular cytokine staining. Whereas neutralizing Abs alone generated minimal frequencies of IL-3–producing cells, addition of certain cytokines induced robust production of IL-3–producing T cells (Fig. 4A). Specifically, considerable numbers of IL-3–producing cells were seen with addition of IL-1α, IL-1β, IL-18, or IL-33. All of these four cytokines belong to the IL-1 cytokine family, indicating a possible common signaling mechanism responsible for driving differentiation to an IL-3–producing subset. Similar experiments were carried out with CD4^+^ T cells from TCR-Tg mice with IL-1α as the selected cytokine, which showed that robust expression of IL-3 was observed with both TCR-Tg models tested (Fig. 4B, Supplemental Fig. 3C). Interestingly, these cells did not coexpress any signature cytokines known to be associated with currently defined functional T cell subsets except for a moderate secretion of IFN-γ (Fig. 4B, 4C, Supplemental Fig. 3C), suggesting relatively little phenotypic and functional overlap with other defined Th subsets.

### Relation of IL-3–producing to GM-CSF–producing CD4^+^ T cells

**FIGURE 5:**
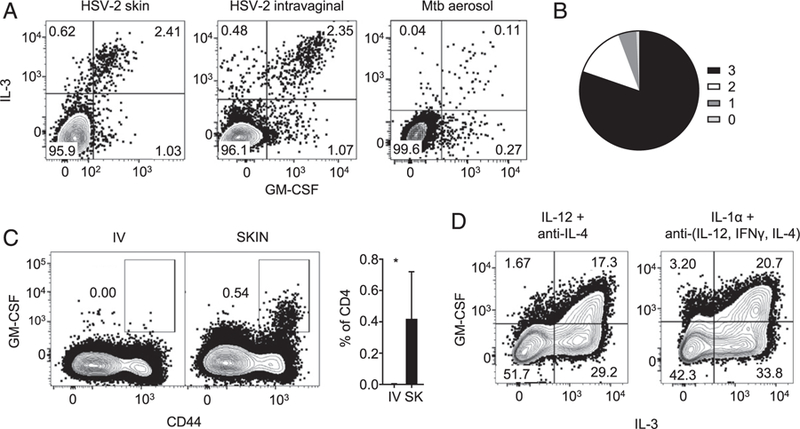
Characteristics of GM-CSF–secreting CD4^+^ T cells. **(A)** Splenocytes from mice infected with HSV-2 cutaneously or intravaginally or infected with *M. tuberculosis* by aerosol route underwent stimu-lation with UV-inactivated HSV-2 or ESAT-6 peptide, respectively, and intracellular cytokine staining. The cells shown are single cells from the lymphocyte gate (FSC/SSC) that are positive for CD4 and CD44 and negative for LIVE/DEAD stain, CD8α, B220, and MHC II. The figure represents results of two independent experiments. **(B)** Splenocytes from mice infected with HSV-2 cutaneously were processed as in (A). Pie chart depicts the fraction of GM-CSF–secreting Ag-experienced CD4^+^ T cells that also secrete cytokines that define multifunctionality. Each shade represents number(s) of additional cytokine(s) secreted. The figure represents results of two independent experiments. **(C)** Splenocytes from mice infected with HSV-2 cutaneously or i.v. underwent stimulation with UV-inactivated HSV-2 and intracellular cytokine staining. Contour plots depict GM-CSF– secreting CD4^+^ T cells obtained postinfection through indicated routes. Bar graphs show the percentage of CD4^+^ T cells that secrete GM-CSF. The figure represents results of two independent experiments. *p < 0.05 for comparisons between i.v. and skin (SK) (Student *t* test). **(D)** Splenocytes from OT-II TCR-Tg mice were cultured in the presence of OVA peptide (OVA_323–339_) and cytokines/Abs as indicated to generate Th1 cells (left) or predominantly IL-3–producing Th cells (right). The contour plot represents coexpression of GM-CSF with IL-3 with the numbers representing the percentage of CD4^+^ T cells for each of the corresponding gates. The figure represents results of two independent experiments.

The IL-3 and GM-CSF genes are closely linked in the human and mouse genome, show similar genomic structures, and share several conserved elements in their 5′ and 3′ flanking regions (35). These two cytokines are shown to be coexpressed in T cells with a few exceptions (34–36). Consistent with these reports, we observed coexpression of IL-3 with GM-CSF in both skin and vaginal HSV-2 infection models and in the *M. tuberculosis* infection model (Fig. 5A). As expected, most GM-CSF–producing cells also expressed cytokines that define multifunctionality (Fig. 5B, Supplemental Fig. 4A). Similarly, restriction to cutaneous route of infection was also seen with GM-CSF–producing CD4^+^ T cells in mice infected with HSV-2 (Fig. 5C). In addition, a subset of Th1 cells generated in vitro–produced GM-CSF (Fig. 5D) and coexpressed cytokines that define multifunctionality (Supplemental Fig. 4B). Finally, a subset of IL-3–producing cells generated under conditions that favor IL-3 production also secreted GM-CSF (Fig. 5D). We noticed that similar to Th1 condition, GM-CSF^+^ IL-3^−^ cells were not generated in this condition. Collectively, these results suggest that the functional characteristics and requirements for generation of GM-CSF–producing CD4^+^ T cells are similar to those of IL-3–producing CD4^+^ T cells. They also indicate substantial although not complete overlap between IL-3– and GM-CSF–producing T cell subsets.

## DISCUSSION

Secretion of signature cytokines, expression of a master transcriptional regulator, and a requirement for a specific cytokine priming environment are considered important criteria for classification of CD4^+^ T cells into distinct functional subsets (37). Our results showed that at least some of these critical features apply to a relatively unstudied population of IL-3–producing T cells, suggesting that they may be a distinct functional and stably differentiated subset. Both in vivo and in vitro, unique conditions were required for the generation of these cells. These included vaccination or infection at barrier surfaces (skin or mucosa) in vivo and the presence of a cytokine belonging to the IL-1 family in vitro. These cells characteristically expressed IL-3 and GM-CSF as signature cytokines, with or without expression of additional cytokines. Our experiments showed that IL-3 secretion was strongly correlated with Th1, Th2, and to a lesser extent Th17 CD4^+^ T cell subsets. Based on these results, we propose a model in which IL-3 and GM-CSF secretion are superimposed on multiple T cell subsets when an infection is introduced at a barrier site such as skin or mucosal membranes. In this scenario, all effector T cell subsets, including Th1, Th2, and Th17 cells, have the ability to additionally express IL-3 and GM-CSF. Although we did not perform any experiments to identify a transcription factor that may be responsible for this expression, we propose that unlike typical master transcription factors that cross-inhibit each other, this transcription factor would be coexpressed with other lineage-specific transcription factors.

The T helper subset we propose might be related to ThGM cells, a previously described potential T helper subset that secretes GM-CSF and IL-3 and is generated in vitro using a combination of IL-7 and anti–IFN-γ Ab (34). However, under our experimental conditions, IL-7 did not result in a significant increase in the production of IL-3 secretion. We also note that another paper that described generation of ThGM cells in vitro achieved it by culturing stimulated CD4^+^ T cells in a medium containing only a combination of Abs against IL-12, IFN-γ, and IL-4 with no added IL-7 (38). This study, however, did not evaluate IL-3 production by these cells. It is possible that different cytokines can preferentially modulate expression of IL-3 and GM-CSF, favoring expression of one or other. For example, Sheng et al. reported that in vitro–derived IL-3–producing CD4^+^ T cells were a subset of GM-CSF– producing cells, whereas the opposite was true in our culture conditions that favored predominantly IL-3–producing cells. It is also important to note that the receptors of GM-CSF and IL-3 are also differentially expressed, with the former expressed heavily on macrophages and monocytes, whereas the latter is more strongly associated with mast cells and basophils. These factors suggest that IL-3 and GM-CSF are not completely redundant in their effects and may also point to distinct functions of CD4^+^ T cell subsets that differentially express these cytokines.

TCR-mediated signaling and cytokine-mediated signaling are considered the major influences on differentiation of naive CD4^+^ T cells into distinct T helper subsets (39). These factors, alone or in combination, may contribute to the generation of IL-3–producing Th cells from naive CD4^+^ T cells. One possibility is that Ag presentation by a specific type of Ag-presenting cells alters the TCR signaling cascade by modifying costimulation signals, resulting in the generation of IL-3–producing Th cells. In this scenario, Ag presentation in the lymph node is mediated by a specific type of dendritic cells resident in the lymph node or migrating from the site of insult at or near the barrier surface. In contrast, after i.v. infection, Ags are presented by cells resident mainly in the spleen or liver. This assumption is in agreement with the previous reports showing qualitative differences in the induced Th cells across various routes of immunization or infection. For example, infection of pathogens through the nasal route supported polarization of naive CD4^+^ T cells into Th17 cells, whereas cutaneous, i.m., or i.v. infection supported Th1 polarization (40–43).

One potential explanation for these results is the differences in the type of cells capturing and presenting Ags at different sites (44–46). Accordingly, priming of CD4^+^ T cells with different Ag-presenting cells has been shown to result in different T helper subsets (47, 48). For example, CD301b^+^ dendritic cells suppress generation of T follicular helper cells and enhance generation of Th2 cells (49, 50). A second possibility is that generation of IL-3–producing Th cells requires a specific cytokine milieu, as supported by our in vitro T helper differentiation experiments. Injury at barrier sites might release certain cytokines that may reach the lymph node by lymphatics, resulting in a unique cytokine milieu at the priming site. Our experiments suggest that IL-1 family cytokines are important components of the cytokine milieu for priming of IL-3–producing T cells. Interestingly, these cytokines are known to be expressed highly by epithelia of the skin or mucosa (29–33), and cytokines derived from the epithelium may influence the differentiation of naive CD4^+^ T cells during Ag priming (51).

Although our own ongoing studies and limited reports indicate only a moderate role for IL-3 in murine models of tuberculosis and HSV (52), there are indications for other possible roles for IL-3–producing CD4^+^ T cells, including in contact hypersensitivity and antiparasite immunity (3, 4). Two studies, to our knowledge, have interrogated the direct role of CD4^+^ T cell–derived IL-3. Ohta et al. (9) showed that adoptive transfer of IL-3–sufficient but not IL-3–deficient CD4^+^ T cells into T cell–deficient mice conferred basophil infiltration and acquisition of tick resistance. Anzai et al. (5) showed that hearts of SCID mice receiving IL-3–sufficient but not IL-3–deficient CD4^+^ T cells accumulated leukocytes causing myocarditis. Interestingly, in both models, IL-3–producing CD4^+^ T cells were generated following Ag delivery through skin. Similar to the latter study, IL-3 has been shown to be deleterious in situations where an unwarranted adaptive immune response leads to disease manifestations such as experimental autoimmune encephalitis (2) and lupus nephritis (6). Thus, the identification of a discrete functional CD4^+^ T cell subset defined by IL-3 secretion extends the potential roles for Ag-specific T cell responses in many types of infectious and inflammatory diseases.

## Supplementary Material

1

## Abbreviations used in this article:

BCG: bacillus Calmette-Guerin
iTreg: induced regulatory T
LLO: listeriolysin O
MHC II: MHC class II
Tg: transgenic
WT: wild-type
