# Supplementary material for: Generation of IL-3–Secreting CD4+ T Cells by Microbial Challenge at Skin and Mucosal Barriers
Source: Immunohorizons. Author manuscript; Available in PMC 2019 Jul 31. (PMC6668923; doi:10.4049/immunohorizons.1900028)
Supplement: 1 [file NIHMS1039874-supplement-1.pdf]

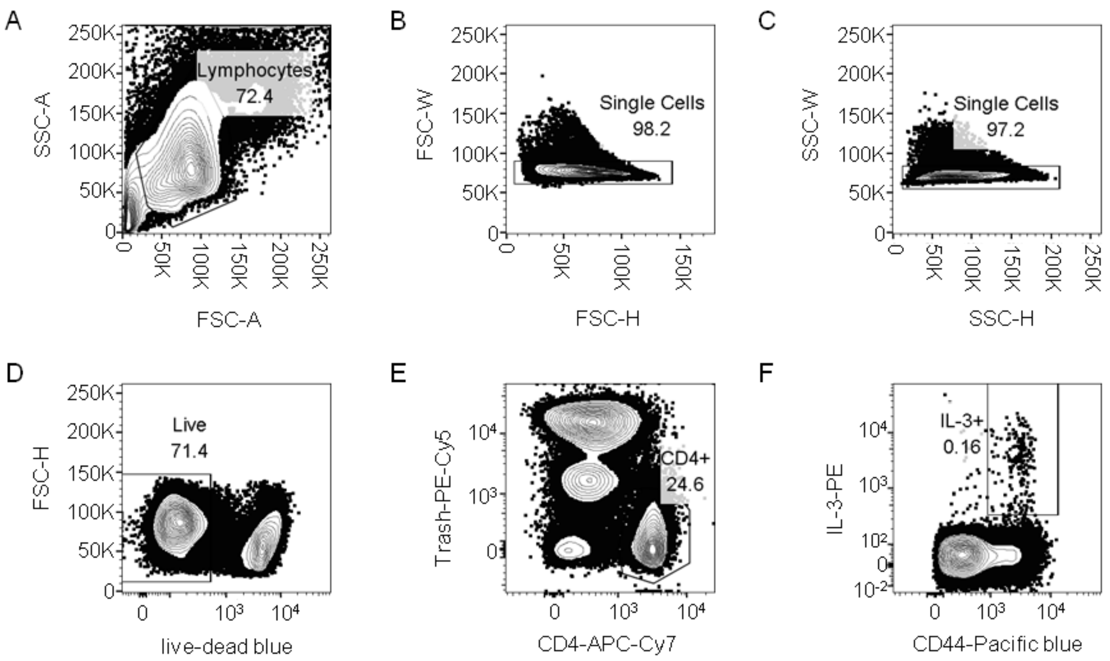

Supplementary Figure. 1 Gating strategy. Gating strategy used to eliminate doublets, dead cells and non-CD4+ T cells are shown. Each contour plot shows the cells gated in the previous plot (A-F) and the numbers indicate the percentage of parent population. Trash cocktail included antibodies to MHCII, CD8α and B220.

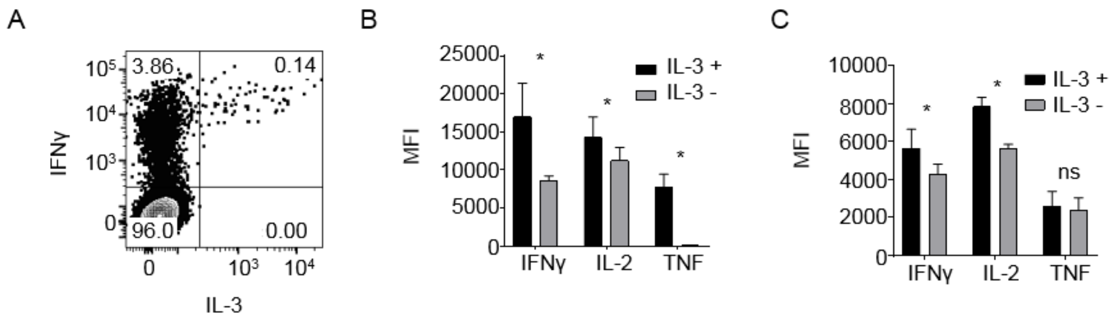

Supplementary Figure. 2 IL-3 secreting CD4$^+$ T cells are multifunctional. (A) Mice were infected with *M. tuberculosis* by the aerosol route. Four weeks later, single-cell suspensions made from mediastinal lymph nodes were re-stimulated with ESAT-6 peptide in the presence of brefeldin A and monensin. Cells were then surface stained, fixed, permeabilized, and stained for intracellular IL-3 and IFNγ. The cells shown are single cells from the lymphocyte gate that are positive for CD4 and negative for Live/Dead stain, CD8α, B220, and MHCII. The figure represents results of two independent experiments. (B) Splenocytes from mice injected with *M. bovis BCG* cutaneously underwent stimulation with *M. tuberculosis* lysate and intracellular cytokine staining. Median fluorescence intensities (MFI) were calculated for each of the cytokines depicted after gating CD4$^+$ T cells based on their IL-3 secretion status. The figure represents results of two independent experiments. (C) Splenocytes from mice infected with HSV-2 by cutaneous route underwent stimulation with UV-inactivated HSV-2 and intracellular cytokine staining. Median fluorescence intensities (MFI) were calculated for each of the cytokines depicted after gating CD4$^+$ T cells based on their IL-3 secretion status. The figure represents results of two independent experiments. **P* < 0.05 for IL-3$^+$ versus IL-3$^-$ cells (Student's t test), and ns indicates not significant.

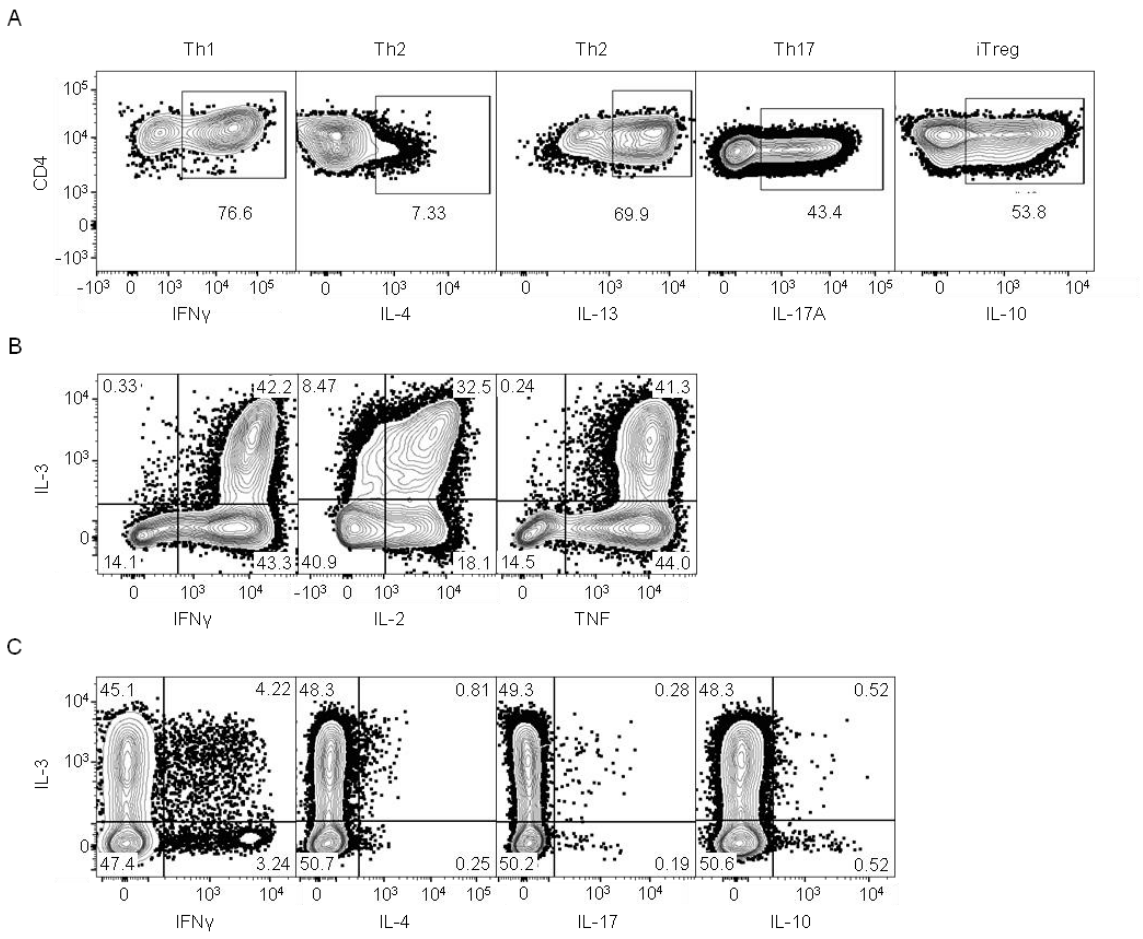

Supplementary Figure. 3 Generation of IL-3 secreting CD4$^+$ T cells *in vitro*. (A) Analysis of CD4$^+$ T cells from cultures prepared as in Figure 3A. The contour plots depict expression of signature cytokines by CD4$^+$ T cells from cultures representing conditions that favor differentiation to the indicated functional subsets. Numbers indicate the percentage of cytokine-positive CD4$^+$ T cells in each gate shown. The figure represents results of two independent experiments. (B) Splenocytes from P25 TCR transgenic mice were cultured in the presence of Antigen 85B$_{240-254}$ peptide, IL-12 and antibody to IL-4 to generate Th1 cells. The samples were processed and the results are displayed as in Figure 3B. The figure represents results of three independent experiments. (C) Splenocytes from P25 TCR transgenic mice were cultured in the presence of Antigen 85B$_{240-254}$ peptide, IL-1α and blocking antibodies to IFNγ, IL-12 and IL-4.

The samples were then processed and the results are displayed as in Figure 4C. The figure represents results of two independent experiments.

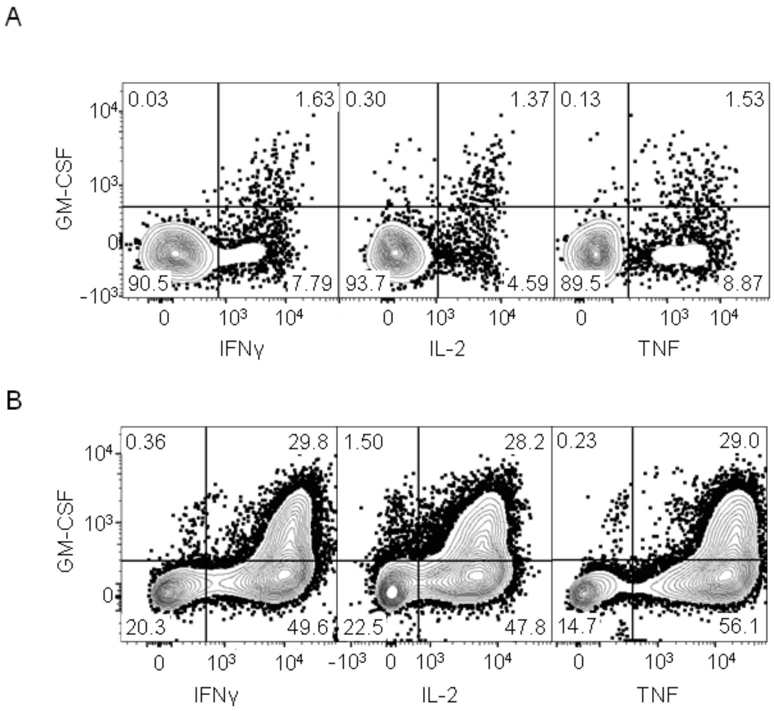

Supplementary Figure. 4 Characteristics of GM-CSF secreting CD4⁺ T cells. (A) Splenocytes from mice infected with HSV-2 by cutaneous route underwent stimulation with UV-inactivated HSV-2 and intracellular cytokine staining. The contour plots depict antigen-experienced $CD44^+$ $CD4^+$ T cells that express IL-3 and cytokines that define multifunctionality. The figure represents results of two independent experiments. (B) Splenocytes from OT II TCR transgenic mice were cultured in the presence of ovalbumin peptide ($OVA_{323-339}$), IL-12 and antibody to IL-4 to generate Th1 cells. The contour plots represent co-expression of GM-CSF with cytokines that define multifunctionality. The figure represents results of two independent experiments.
